# Contributions of intestinal protists on the human gut landscape through the lens of *Entamoeba* spp.

**DOI:** 10.1128/msphere.00011-26

**Published:** 2026-06-09

**Authors:** Marienela Y. Heredia, Laura J. Knoll

**Affiliations:** 1 Department of Medical Microbiology and Immunology, University of Wisconsin-Madison 5228, Madison, Wisconsin, USA; 2 Cellular & Molecular Pathology Graduate Program, University of Wisconsin-Madison 5228, Madison, Wisconsin, USA; The University of Auckland, Auckland, New Zealand

**Keywords:** *Entamoeba*, host-pathogen interactions, gut ecosystem, microbial diversity

## Abstract

Intestinal protists represent an underappreciated yet functionally significant component of the human gut microbiome. Historically dismissed as parasites or transient contaminants, many of these microbial eukaryotes, particularly *Entamoeba* spp., are now recognized as integral to gut ecosystem function and host immune homeostasis. This review examines the complex roles of *Entamoeba* spp. in the mammalian gut, positioning them as dynamic microbiome “landscapers” that influence host-pathogen interactions, immune tone, and microbial diversity. We explore the evolutionary adaptation of *Entamoeba* to the gut’s anaerobic and immunologically active environment, highlighting both pathogenic (*E. histolytica*) and non-pathogenic species (*E. dispar*, *E. coli*) and their distinct immunomodulatory strategies. Special attention is given to the host immune responses shaped by *E. histolytica*, including inflammasome activation, macrophage polarization, and suppression of protective type-2 responses. The review also details *Entamoeba*’s interactions with the gut microbiota, emphasizing their capacity for selective bacterial predation, disruption or enhancement of microbial community structure, and synergistic or antagonistic relationships with commensals and pathogens alike. Methodological challenges in protist detection, genome annotation, and cultivation are discussed, alongside promising advances in sequencing, host DNA depletion, and animal modeling. Taken together, current evidence reframes *Entamoeba* spp. not as mere pathogens but as key ecological players whose presence can signal resilience or susceptibility within the gut ecosystem. Understanding the context-dependent functions of intestinal protists may offer new insights into microbial therapeutics, immune modulation, and disease prevention strategies.

## INTRODUCTION

## INTESTINAL PROTISTS ARE THE OVERLOOKED “LANDSCAPERS” OF THE GUT MICROBIOME

In recent years, the human gut microbiome has emerged as a focal point of biomedical research, with growing recognition of its influence on host health, disease susceptibility, and immune regulation (1, 2). The gut microbiome represents a diverse, cross-kingdom ecosystem harboring bacteria, fungi, protists, helminths, and viruses. These organisms interact with their host and with each other in dynamic ways and play countless roles in shaping both host immunity and gut metabolism. Despite the diversity of the gut microbial ecosystem, microbiome research has largely skewed toward understanding the composition and roles of bacterial communities in this niche. As a result, resident gut microbial eukaryotes often remain overlooked. This inattention is especially true for intestinal protists, which are generally found in lower abundances among individuals living in industrialized communities compared to non-industrialized populations due to modern medical practices, increased hygiene awareness, and reduced environmental exposure to microbial diversity (3, 4). Indeed, medical and scientific paradigms shaped by Western biomedical education have traditionally cast intestinal protists in a parasitic light, with clinical focus limited to enteric disease-causing species such as *Cryptosporidium parvum*, *Giardia intestinalis*, and *Entamoeba histolytica*. This educational approach results in an inherent bias among healthcare providers against all intestinal protists regardless of their pathogenic potential or a patient’s current health status (5).

Evolutionary history paints a more complex picture of intestinal protists. The mammalian gut is a unique ecological niche characterized by several environmental constraints, including low oxygen concentrations, fluctuating nutrient availability and pH levels, high cell turnover, and mechanical force caused by peristalsis. In addition, resident immune cells protect unwanted microbial pathogens from invading and disrupting gut function. This restrictive microenvironment has driven deep co-adaptation between intestinal protists and their hosts. Non-pathogenic protists are found colonizing healthy individuals across the globe, particularly in non-industrialized settings (3). Far from being mere pathogens or passive bystanders, these protists function as ecosystem engineers within the gut, particularly by modulating intestinal barrier permeability, training the host immune system, maintaining microbial diversity, and offering protection against enteric pathogens (6–11). By establishing these symbioses, intestinal protists may be better understood as microbiome remodelers, or landscapers of the gut environment that shape its terrain in both subtle and profound ways.

Importantly, this “landscaper” function is not restricted to a single protist lineage. A growing body of work across diverse intestinal protists supports a broader role for these organisms as ecological modulators of the gut environment. For example, colonization with *Blastocystis* spp. has been consistently associated with increased bacterial diversity and shifts in microbial community structure, often correlating with markers of gut health and host dietary choices (12–15). Similarly, *Giardia intestinalis* infection has been shown to alter nutrient availability, disrupt mucus architecture, and reshape microbial metabolic output, even in asymptomatic contexts (16, 17). The commensal protist, *Dientamoeba fragilis*, has also been linked to distinct microbiome configurations, further supporting a role in structuring microbial communities (18, 19). In addition, emerging work on gut-associated *Tritrichomonas* spp. demonstrates direct inter-kingdom interactions with bacterial populations and host tissues resulting in modulation of host epithelial signaling, gut mucosal immune tone, and protist metabolic reprogramming (20–27). Collectively, these findings reinforce a model in which intestinal protists act as active participants in shaping the gut ecosystem, influencing microbial composition, resource landscapes, and host responses in ways that extend beyond classical definitions of parasitism.

## METHODOLOGICAL HURDLES TO STUDYING INTESTINAL PROTISTS

To date, the parasitology field has heavily relied on human population-level surveys in endemic regions, which provide important prevalence data but offer limited mechanistic insight into how these organisms interact with their host or other members of the microbiome. Molecular approaches such as 16S rRNA metabarcoding, optimized for bacterial detection, are ill-suited to profiling microbial eukaryotes. While 18S rRNA-based eukaryome analyses exist, they are complicated by the presence of host and dietary plant DNA, which can mask relatively low-abundance protist signals. Recently, a novel metabarcoding approach involving effective depletion of host 18S sequences has been developed, making taxonomic analyses of host-associated eukaryotic communities much more feasible and reliable (28). Metagenomic sequencing also offers a promising alternative to amplicon-based sequencing; however, the higher relative abundance of bacteria compared to eukaryotes in the microbiome remains a challenge. Recent advances in this area have also been achieved by leveraging flow cytometry and optimized DNA extraction approaches to selectively recover and lyse eukaryotic cells from a mixed microbiome sample (29). Additionally, recent developments of metagenomic assembly pipelines tailored to microeukaryote identification have drastically increased the utility of metagenomic sequencing for eukaryome analyses (30, 31). Still, the scarcity of fully sequenced and annotated protist genomes hinders the development of robust bioinformatic pipelines for classification, assembly, and functional analysis of intestinal protists.

Experimental research on host-protist interactions is also limited, as current culture systems are complicated and highly species-specific. Because protists are so phylogenetically diverse, there is no all-in-one media that can support growth of all intestinal protists. Current culturing systems for some model intestinal protists can be fastidious, requiring technical expertise, reagent lot testing, and in some cases, supplementation with feeder bacteria to support protist growth (32). These systems are ideal for investigating key elements of protist biology but are difficult to apply to studies involving host-protist interactions. As with many microbiome-associated bacterial species (33), many intestinal protists have both nutritional and environmental requirements for survival that are generally incompatible with established mammalian cell lines. As such, more complex cell culture systems, such as intestinal organoids, explants, or gut-on-a-chip models, may be required to conduct physiologically relevant co-culture experiments (34). In some cases, the development of human-translatable, *in vivo* infection models is also limited by host species specificity and an inability to replicate the complexities of entire protist life cycles. Host species specificity can depend on a number of factors, including host-specific colonization factors and gut microbiome composition across heterologous animal models. Since intestinal protists have largely co-evolved alongside complex bacterial communities, species-specific differences between human and animal microbiota and their metabolites likely exhibit profound effects on protist colonization dynamics and interactions with the host immune system, thus limiting the direct translatability of *in vivo* findings.

Despite these obstacles, the past decade has seen considerable progress in redefining the roles of gut protists within the human microbiome. This review will focus on a major genus of human-associated intestinal protists of major biomedical relevance: *Entamoeba* spp. We explore their colonization dynamics, immunomodulatory roles, and influence on microbial community structure. We also review current experimental approaches, highlight key discoveries, and propose future research directions aimed at disentangling the complex, often context-dependent contributions of *Entamoeba* spp. to gut health and immune homeostasis.

## *ENTAMOEBA* SPP. AND THE MAMMALIAN GUT

### The *Entamoeba* life cycle and its relevance to human health

Amoebae in the genus *Entamoeba* are widespread across nature, with many species adapting to specific hosts and environmental niches. The most well-known *Entamoeba* species due to its relevance to human health is the intestinal parasite, *Entamoeba histolytica. E. histolytica* infects approximately 500,000 to 1 million people globally per year; however, this is thought to be a vast underestimate due to underreporting of asymptomatic to mild cases of infection (35, 36). *E. histolytica* is microscopically identical to its lesser studied and non-pathogenic relative, *Entamoeba dispar*, also leading to mischaracterization or unclear diagnoses between the two species. A recent meta-analysis of *Entamoeba* prevalence data in the Americas determined a pooled prevalence of around 9% (37). In addition, *E. histolytica/E. dispar* infection disproportionately affects men who have sex with men (MSM) worldwide. A Sydney cohort study found that the prevalence of *Entamoeba* spp. among MSM was approximately 11.1%, compared to 0% in non-MSM controls (38).

While *E. histolytica* typically remains non-invasive in the human intestinal tract, well confined by protective mucus barriers, approximately 10% of *E. histolytica* infection cases lead to development of amebiasis, resulting in 55,000–100,000 deaths annually (35). Notably, *E. histolytica* continues to be the only parasite among *Entamoeba* spp. associated with extraintestinal disease (39). Amebiasis can present itself in several different ways, with symptoms ranging from amebic dysentery, amebic colitis, and dissemination to the liver, where it induces amebic liver abscess formation (36). In rarer cases, *E. histolytica* may also disseminate into the lungs and brain to cause death (40). To date, the host factors contributing to invasive disease susceptibility in this subset of *E. histolytica* cases remain poorly understood; however, malnutrition and co-infection with the bacterial pathogen, *Clostridioides difficile*, among susceptible individuals are thought to play a role (41, 42).

Infection and transmission of *Entamoeba* spp. requires the ingestion of infectious, environmentally resistant cysts found in fecal contaminated food and water sources. These cysts undergo stage interconversion via a process called excystation upon reaching the distal small intestine (43). Through this process, amoebae exit their chitin-rich cyst wall and become motile, metabolically active trophozoites. These trophozoites begin to multiply via binary fission and colonize the colon (43). *Entamoeba* thrives in the anaerobic environment of the colon, preying on resident bacteria, degrading host mucin, and consuming available iron to sustain their dietary needs (44). Upon receiving signals thought to involve high densities of amoebae and nutrient limitation, trophozoites aggregate together to reform cysts in a process called encystation (45). These newly formed cysts are then excreted in the feces of infected hosts, contaminating the environment and repeating the fecal-oral infection cycle. While many of the specific molecular and cellular mechanisms that govern these stage interconversions remain undefined, significant insight has been gained using the reptile-specific pathogen, *Entamoeba invadens*, as an *in vitro* stage interconversion model (46–48). More recently, an *in vitro E. histolytica* encystation system has been generated, further opening other avenues of investigation into the life cycle and transmission of this human parasite (45).

Despite the relevance of *Entamoeba* infection to human health, animal infection models of *Entamoeba* spp. are scarce. The most common infection modality with *E. histolytica* involves injection of cultured *E. histolytica* trophozoites in surgically exposed mouse ceca or artificial colonic loops (49). This model allows for the study of *E. histolytica* pathogenesis, specifically in the context of amebic colitis. The formation of amebic liver abscesses can also be recapitulated via intraportal inoculation of trophozoites into rodents such as mice, guinea pigs, or hamsters (50). While these models have greatly enhanced our understanding of *E. histolytica* infection and pathogenesis *in vivo*, they bypass the natural fecal-oral route of infection and require extensive training and expertise to perform. An *E. histolytica* oral infection study has been conducted in rats treated with cimetidine to inhibit the production of stomach acid (51). Recently, a fecal-oral transmission model of infection has been developed using the mouse commensal species, *Entamoeba muris* (52). This model recapitulates the entirety of the *Entamoeba* life cycle in mice and allows for the study of non-pathogenic *Entamoeba* infections. Indeed, 90% of *E. histolytica* cases involve asymptomatic infection, and other human-associated non-pathogenic *Entamoeba* spp., such as *E. dispar*, *E. polecki*, *E. hartmanni*, and *E. coli*, have been described, even in the same host (53). In the Americas, *E. coli* was found to be the most widely distributed and prevalent *Entamoeba* spp. colonizing humans, with an incidence of 21% (37). In a eukaryome characterization study carried out among children in the Central African Republic and Madagascar, various *Entamoeba* species were detected: *E. dispar/histolytica* present in 21% of samples, *E. coli* and *E. hartmanni* in 22% of samples, *E. polecki* in 8% of samples, and *E. bovis* in 10% of samples (53). Approximately one-quarter of samples tested exhibited co-infection with more than one of these *Entamoeba* species, suggesting that co-infection with both pathogenic and non-pathogenic *Entamoeba* species may be common. Given these findings and the positive correlation between *Entamoeba* infection and colonization with *Blastocystis*, another widespread human intestinal protist, inter-protist interactions within the human gut may warrant a novel and exciting area for further research (53).

### Host immune responses to *Entamoeba* spp.

**Fig 1:**
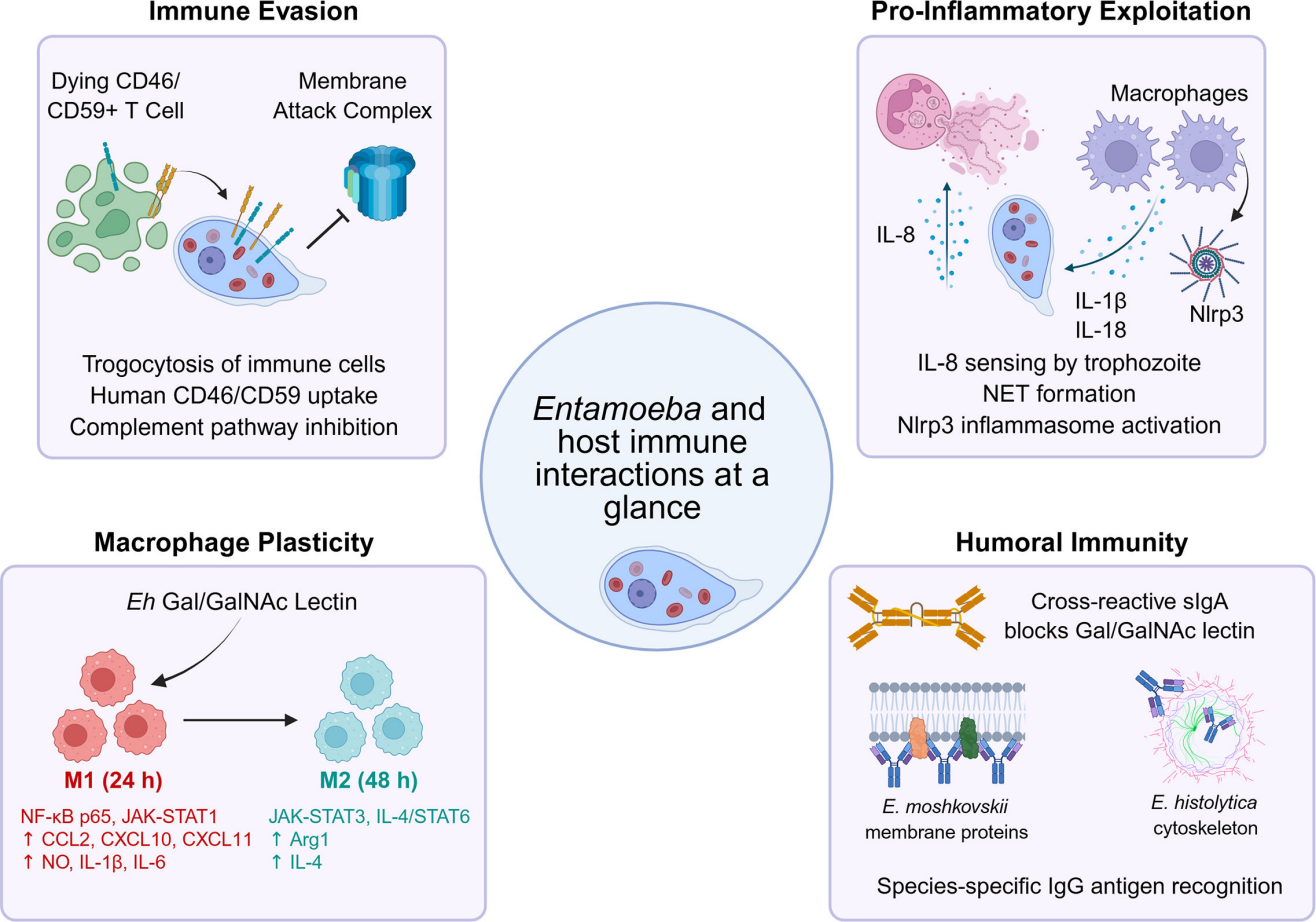
*Entamoeba*-host interactions through an immunological lens. Trophozoite-mediated immune evasion through trogocytosis and human complement regulatory protein acquisition facilitates persistence within the gut. Parasites sense and migrate toward IL-8-rich inflammatory sites, where neutrophil extracellular traps (NETs) and inflammasome activation by macrophages sustain a pro-parasitic, highly inflammatory microenvironment. Time-dependent modulation of macrophage polarization supports early inflammation and later tissue repair, promoting longer parasitic colonization. Humoral responses to Gal/GalNAc lectin confer protection via secretory IgA (sIgA) and species-specific IgG, which can recognize either membrane or cytoplasmic antigenic proteins in related *Entamoeba* spp. Image created with Biorender.com.

Interactions between the host immune system and *Entamoeba* spp. have been best characterized through the lens of *E. histolytica* due to its pathogenic potential in humans. *E. histolytica* is uniquely adapted to evade the host innate immune system, contributing to its ability to colonize the human gut for extended periods of time (54). One mechanism used by the parasite to avoid detection is via trogocytosis, or nibbling, of host immune cells (55). This mechanism was first described during co-incubation of *E. histolytica* trophozoites with human Jurkat T cells (55). Trogocytosis not only allows *E. histolytica* to trigger host cell death in a NOX4-dependent manner (56) but also allows the parasite to uptake and display host immunomodulatory proteins like the complement regulatory proteins, CD46 and CD59, on their surface to avoid detection by the host complement system (57, 58) (Fig. 1). In addition to immune evasion mechanisms, differences in humoral immune responses can also affect persistence of *Entamoeba* infection. Infected individuals carry high levels of Gal/GalNAc lectin-specific IgA, which was protective against reinfection in both a cohort of susceptible Bangladeshi children and patients recovering from amebic liver abscesses (59–61). Cross-species protection by these lectin-specific IgA was observed in subsequent infection with *E. dispar* (60). Immunization conducted on BALB/c mice with a combination of *E. histolytica* and *E. moshkovskii* produced a significantly higher IgG antibody response than immunization with either species alone (62). *E. moshkovskii* immunization also led to a significantly more robust and longer-lasting antibody response than did immunization with *E. histolytica*. This species-specific effect in antibody response was likely due to differences in antigenic proteins between species; while 89% of *E. histolytica*-specific monoclonal antibodies targeted cytoplasmic and cytoskeletal proteins, 73% of *E. moshkovskii-*specific monoclonal antibodies targeted membrane proteins (62) (Fig. 1).

Inflammation is central to *E. histolytica* survival and pathogenesis in the host. In fact, a hallmark of *E. histolytica* infection involves neutrophil recruitment and release of neutrophil extracellular traps (NETs), contributing to an ongoing, hyperinflammatory environment the parasite thrives in (63). Further evidence for this preference lies in the ability of *E. histolytica* to migrate toward inflammation sites via sensing of IL-8 through a trophozoite membrane protein resembling the human chemokine receptor, CXCR1 (64). *E. histolytica* infection also increases the production of bioactive IL-1β and IL-18 by hyperactivating macrophages in the lamina propria via caspase-4/1 activation and recruitment of the NLRP3 inflammasome (65) (Fig. 1). Macrophages play a vital role not just in parasite-induced inflammation but also in persistence of *E. histolytica*. A recent study demonstrated that during early infection stages, the C-fragment of the *E. histolytica* Gal/GalNAc lectin induces M1 macrophage polarization via the activation of the NF-ΚB p65 and JAK-STAT1 signaling pathways after 24 h post-infection (66). This response was characterized by increased nitric oxide production and increased transcription of chemokines CCL2, CXCL10, and CXCL11, and the pro-inflammatory genes, IL-1β, and IL-6. By 48 h post-infection, this same C-fragment of the Gal/GalNAc lectin also induced M2 macrophage polarization via the activation of JAK-STAT3 and IL-4-STAT6 signaling pathways, leading to increased arginase activity and IL-4 production (66) (Fig. 1).

**Fig 2:**
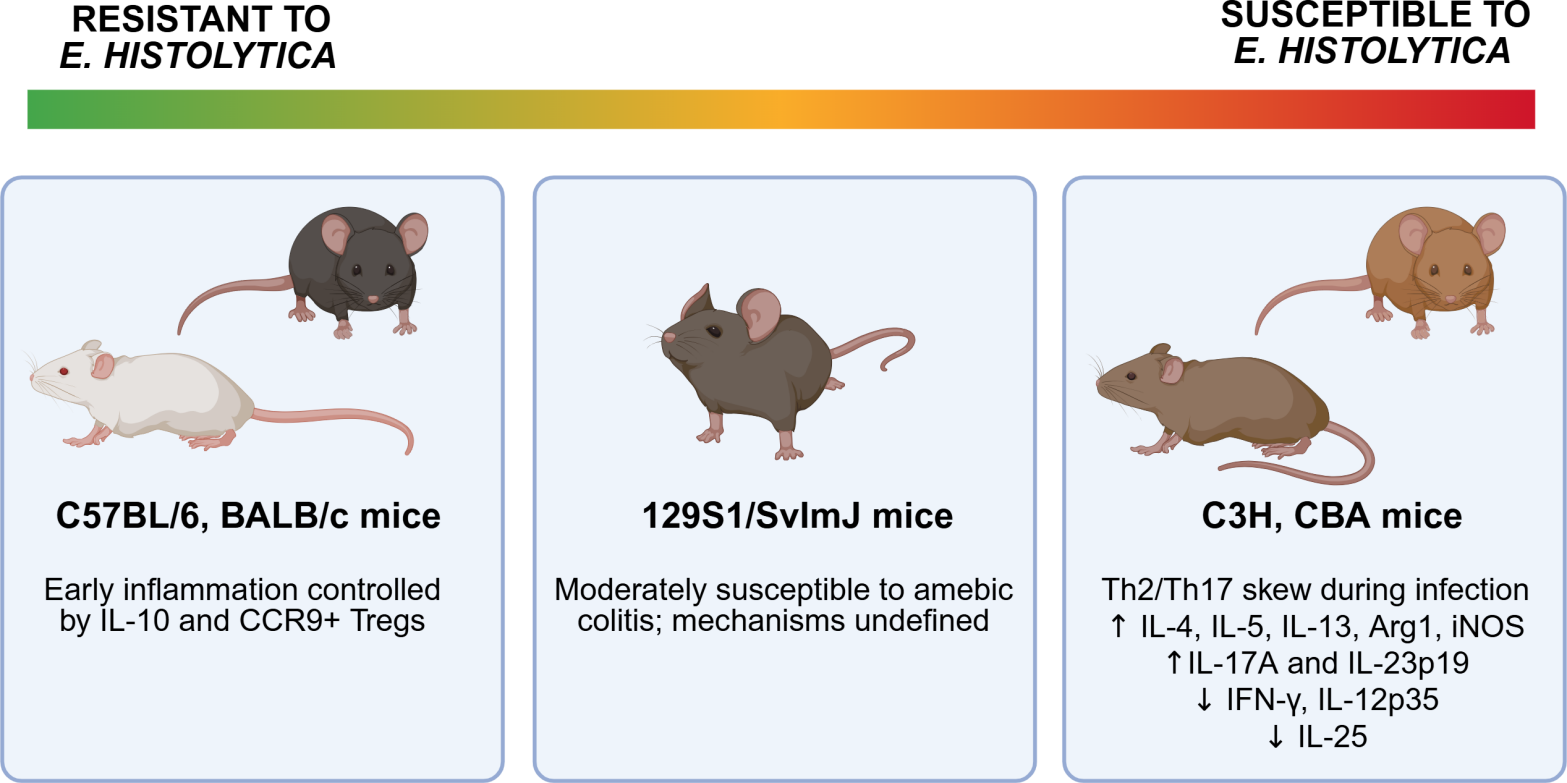
Mouse genetic backgrounds confer differing degrees of resistance and susceptibility to *E. histolytica* infection. Mouse strain differences alter immune responses to *E. histolytica* infection via intracecal injection. Image created with Biorender.com.

Along with *in vitro* infection systems, rodent infection models have also played a vital role in understanding *E. histolytica* pathogenesis. However, both disease severity and immune responses to *E. histolytica* in mice are highly dependent on their genetic background (Fig. 2). C57BL/6J and BALB/c mice are naturally resistant to *E. histolytica* infection via direct cecal injection (67); however, a recently published model of fecal-oral *E. muris* colonization in BALB/c mice does exhibit colitis-like pathology driven by early CD4^+^ T cell infiltration and recruitment of FoxP3^+^ Tregs in the colon (68). Studies investigating the natural resistance of C57BL/6J mice to *E. histolytica* found that resistance to infection was dependent on the production of the immunosuppressive cytokine, IL-10 (69). This protection may be conferred by CCR9^+^ Tregs, as CCR9^−/−^ mice infected with *E. histolytica* had no detectable cecal IL-10 levels and instead exhibited chronic intestinal inflammation associated with the overexpression of IFNγ, TNFα, IL-4, IL-6, and IL-17 (70). In addition, these mice showed delayed recruitment of CD4^+^CD25^+^FoxP3^+^ T cells to the cecal epithelium and lamina propria, indicating a defect in early regulation of the inflammatory response to *E. histolytica* (70). Importantly, IL-10 is a major contributor to the development of non-infectious colitis, as IL-10^−/−^ mice develop spontaneous, chronic enterocolitis and, thus, serve as a major model of inflammatory bowel disease pathogenesis (71). These studies indicate that dysregulated colonic inflammation is a key driver of *E. histolytica* pathogenesis.

Unlike the naturally resistant C57BL/6J and BALB/c mice, 129S1/SvImJ mice are moderately susceptible to *E. histolytica* infection by cecal injection, while CBA/J and C3H mice are highly susceptible (72). CBA/J and C3H mice develop non-healing chronic intestinal lesions and higher intestinal inflammation scores during *E. histolytica* infection (67, 72). This phenomenon appears to be driven by T cells, as *E. histolytica*-infected C3H mice carried lower parasite burdens and decreased inflammation upon depletion of CD4^+^ T cells (67). In agreement with these studies, both T cell-depleted and T cell-deficient BALB/c mice are unable to support persistent colonization by *E. muris* (68). In CBA/J mice, *E. histolytica* has been shown to rapidly induce a mixed Th2/Th17 mucosal immune profile, characterized by increased expression of IL-4, IL-5, IL-13, arginase-1 (Arg1), iNOS, IL-17A, and IL-23p19 in the ceca and mesenteric lymph nodes (72). These cytokine changes were accompanied by a decrease in the expression of Th1 cytokines, IFN-γ and IL-12p35, after 3 days post-infection, while IL-10 remained mostly unchanged. Anti-IL-4 therapy in *E. histolytica*-infected CBA/J mice led to parasite clearance via restoration of IFN-γ production (72). This finding supported clinical data demonstrating higher serum IL-4 levels in patients with invasive amebiasis as well as a protective effect for higher IFN-γ levels in children (73, 74).

The classical, type-2 anti-parasitic immune response is best characterized by secretion of IL-25 and IL-33 by tuft cells and intestinal epithelial cells, respectively. Both IL-25 and IL-33 are sensed by type-2 innate lymphoid cells (ILC2), which subsequently produce IL-4, IL-5, and IL-13 (75). These cytokines are the primary effectors of type-2 immunity, signaling recruitment of eosinophils, differentiation of naïve, CD4^+^ T cells into Th2 cells, and expansion of both intestinal tuft cells and goblet cells via differentiation of Lgr5^+^ stem cells in the crypts (75). Surprisingly, recent studies have demonstrated that IL-25 production is suppressed during amebic colitis both in human patients and in CBA/J mice infected with *E. histolytica*, while TNFα is increased in colon biopsies of human amebic colitis patients (76). Treatment with recombinant IL-25 contributed to parasite clearance and reduced colon pathology, and the depletion of eosinophils using anti-SiglecF monoclonal antibody abrogated this protective response (76). Treatment of *E. histolytica*-infected CBA/J mice with recombinant IL-33 ameliorated disease severity and parasite burden in the colon and cecum, respectively (77). The protective effect of IL-33 appears unique to amebic colitis, as IL-5^+^ ILC2 activated by IL-33 exacerbated the formation and severity of amebic liver abscesses in *Rag2*^−/−^ mice via increased recruitment of neutrophils and eosinophils (78). Taken together, these findings support a model in which a type-2 immune response driven by IL-25 and IL-33 production is protective specifically against intestinal *E. histolytica* infection, and dysregulation of this response by the parasite may contribute to the development of amebic colitis.

While much insight has been gained from studying interactions between host immunity and *E. histolytica*, little is known about the immunomodulatory effects of non-pathogenic *Entamoeba* spp. on gut mucosal immunity. *E. dispar*, unlike *E. histolytica*, does not induce NET formation, pointing to an inherent difference in virulence and pathogenic potential between the two species (79). As non-pathogenic *Entamoeba* spp. continue to be detected in healthy individuals worldwide, unraveling their immunological footprints could provide critical insight into the spectrum of host-protist interactions and their relevance to gut health and disease resilience.

### *Entamoeba* spp. and the gut microbiome

As predators of native gut bacteria, *Entamoeba* spp. have the capacity to alter the composition and functionality of the gut microbiome. These compositional changes induced by *Entamoeba* colonization are largely dependent on the region, host health status, and diet. A recent study characterizing intestinal protist effects on the gut microbiomes of children in Colombian daycares found that gut colonization with the non-pathogenic species, *Entamoeba coli*, increased microbiome richness and was positively correlated with enrichment in bacteria of the genera, *Akkermansia, Coprococcus*, and *Alistipes* (80). Comparative microbiome studies conducted among rural African populations with differing modes of subsistence found that *Entamoeba* colonization correlated with increased alpha diversity, decreased beta-diversity, and significantly altered microbial composition (81). Increased alpha-diversity has been observed among individuals colonized with other non-pathogenic intestinal protists, such as *Blastocystis*, *Endolimax nana*, and *Dientamoeba fragilis* (18, 80, 82–84), while decreased alpha diversity has been reported among individuals infected with the parasite, *Cryptosporidium* spp. (85). In addition, the reduced beta-diversity observed among *Entamoeba*-colonized individuals may indicate convergence toward a shared microbial community structure, consistent with a potential ecosystem-shaping role for *Entamoeba* and, in turn, intestinal protists. *Entamoeba*-positive individuals in this study also demonstrated distinct microbial composition signatures that have been implicated in inflammatory bowel disease and autoimmune disorders (81). Specifically, the presence of *E. histolytica/dispar* significantly correlated with an enrichment of the bacterial taxon, Clostridiales Ruminococcaceae, which has been found to be underrepresented in individuals with inflammatory bowel disease (81, 86). Additionally, colonization by *E. histolytica/dispar* correlated with decreased prevalence of *Prevotella copri* and Fusobacteria, which have been associated with the development of rheumatoid arthritis and colorectal cancer (87–89).

Gut microbiome changes can also be associated with amebiasis. A recent study conducted in Japan comparing gut microbiome changes in patients with invasive or asymptomatic *E. histolytica* infection found that beta diversity, a metric of bacterial composition, differed significantly between both populations. Asymptomatic individuals carried a more uniform microbiome (90). OTU analyses of these patient microbiomes revealed that the proportion of Streptococcaceae was significantly lower in asymptomatically infected individuals compared to patients with invasive disease. In addition, the proportions of Ruminococcaceae, Coriobacteriaceae, and Clostridiaceae, which have been associated with beneficial roles in the microbiome, were significantly higher in asymptomatically infected individuals (90). At the species level, *Collinsella aerofaciens* was significantly enriched in asymptomatic individuals, while *Streptococcus salivarius* and *Streptococcus sinensis* were significantly lower (90). Studies conducted in BALB/c mice infected with *E. muris* have also described notable shifts in bacterial microbiome composition during colitis; *E. muris* infection reduced the abundance of 18 different bacterial species, 10 of which belong to the phylum, Firmicutes (68). Taken together, these findings suggest that a loss of potentially beneficial bacteria and an increase in potentially exacerbating bacteria may be a contributor to the development of invasive disease.

Bacterial-amoebic interactions have also been studied *in vitro*, providing valuable insights into these complex, and often protective, cross-kingdom interactions. Co-incubation experiments of *E. histolytica* with a diverse mixture of gut bacterial species found that *E. histolytica* preferentially phagocytosed commensal bacteria of the families, Lactobacillales, Erysipelotrichales, Clostridales, and Bifidobacteriales (91). Further analysis showed that *E. histolytica* displayed a significant preference for *Lactobacillus ruminus*, a resident member of a healthy human gut microbiome (91). In the context of the human gut, bacterial communities rarely live as planktonic, free-living cells. Rather, they exist in complex microbial communities called biofilms, which confer unique protection against environmental insults, host immune attack, and antimicrobial drug resistance (92). A recent study using *Bacillus subtilis* as a model demonstrated that *E. histolytica* is adapted to predate on bacterial biofilms in the gut despite the protective nature of this microbial lifestyle via secretion of cysteine proteases in a process called digestive autophagy (93). These proteases allow the parasite to both adhere to and break down the bacterial biofilm extracellular matrix. These interactions appear to only benefit the amoebae, as the adhesion to the bacterial biofilms conferred protection to *E. histolytica* against oxidative stress, while secreted amebic products restored antibiotic sensitivity to the biofilms (93).

*Entamoeba* biology is impacted not only by direct interactions with other bacteria but also by their metabolites. Co-incubation of *E. histolytica* with enteropathogenic *Escherichia coli* O55 led to enhanced resistance of the parasite to oxidative stress via *E. coli*-derived oxaloacetate (94). Furthermore, exposure to live *E. coli* O55 significantly enhanced superdiffusive motility in the parasite, leading to broader dispersal and more extensive cell destruction (95); however, whether this phenotype was also induced by an *E. coli*-derived metabolite remains unclear. Short-chain fatty acids produced by commensal bacteria have also been shown to differentially induce stage interconversion in *Entamoeba* spp. in a host-specific manner. Butyrate prevented *in vitro* encystation of *E. invadens* yet exerted no significant effect on encystation of *E. histolytica* (45, 96). Meanwhile, acetate and propionate induced earlier *in vitro* encystation in *E. histolytica* (45). These findings suggest that host-specific bacterial microbiomes and their metabolites can exert unique biological effects on *Entamoeba* spp.

Taken together, these studies reveal a nuanced ecological role for *Entamoeba* spp. in shaping the gut microbiome and influencing host health. Through selective predation, modulation of microbial diversity and exploitation of biofilm communities, *Entamoeba* spp., including both pathogenic and commensal strains, emerge as potent remodelers of microbial ecosystems. Their interactions with gut bacteria appear to be both strategic and context-dependent, with colonization outcomes shaped by host geography, health status, and microbial milieu. These findings not only expand our understanding of *Entamoeba*-microbiota crosstalk but also raise important questions about how such interactions may tip the balance between homeostasis and disease.

## CONCLUSIONS

The mammalian gut represents one of the most complex and dynamic microbial ecosystems in nature, yet the full diversity of its eukaryotic members has only recently begun to be recognized for its contributions to gut homeostasis and disease. As this review underscores, *Entamoeba* spp., long relegated to the realm of parasitology and pathogenesis, are emerging as nuanced players within the microbiome landscape. Far from being uniformly pathogenic, many *Entamoeba* species colonize healthy individuals without clinical consequence and, in some contexts, may even contribute to intestinal homeostasis and microbial diversity. These findings challenge the long-standing assumptions that protists are merely harmful invaders and instead support a broader ecological framework in which *Entamoeba* spp. can act as both disruptors and remodelers of the gut terrain.

Indeed, the duality of *Entamoeba* spp. as both potential pathogens and commensals reflects their finely tuned adaptations to a host environment shaped by immunological, nutritional, and microbial constraints. The capacity of *E. histolytica* to subvert innate immune responses, exploit host inflammation, and strategically predate on resident microbiota exemplifies its evolutionary specialization. Yet its close relatives, such as *E. dispar*, *E. coli*, and *E. moshkovskii*, appear to lack these virulence traits and instead correlate with increased microbial richness and stability in various human populations. This functional divergence among *Entamoeba* species raises critical questions about the molecular determinants of pathogenicity and the contextual factors that govern disease emergence versus tolerance.

Host immunity clearly plays a decisive role in mediating these outcomes. From the hyperinflammatory environments that enable *E. histolytica* invasion to the protective IL-25- and IL-33-mediated signaling cascades disrupted during amebic colitis, the host response is not simply a defense mechanism but a complex regulatory force that shapes the protist’s ecological niche. Mouse models with differing genetic susceptibility have revealed that cytokine tone, macrophage polarization, and regulatory T cell dynamics significantly influence infection trajectory. These insights are essential for developing targeted therapeutics and vaccine strategies that reinforce protective immune pathways while avoiding collateral inflammation.

Equally important are the microbial communities in which *Entamoeba* resides. The bidirectional interactions between *Entamoeba* and gut bacteria, ranging from selective phagocytosis of commensals to synergistic modulation of biofilm communities, suggest that protist colonization cannot be understood in isolation. Rather, it must be considered within the broader network of microbial competition, cooperation, and host-mediated feedback. Notably, the microbiota composition in asymptomatic vs invasive *E. histolytica* infections differs in consistent, measurable ways, hinting at microbiome configurations that may either buffer against or potentiate disease. These associations may also extend to co-colonizing protists, such as *Blastocystis* and *Dientamoeba fragilis*, which are frequently detected among people carrying intestinal protists and are geographically widespread (7). Indeed, emerging data point to possible inter-protist synergism between *Entamoeba* spp. and *Blastocystis* (53).

Despite these advances, significant gaps remain in our understanding of *Entamoeba* spp. Methodological limitations in detecting, classifying, and experimentally manipulating *Entamoeba* continue to hinder progress. As a tetraploid organism, gene editing and gene knockout studies are not yet genetically feasible although recent developments using RNA interference and other genetic tools are beginning to open more investigative avenues toward loss-of-function studies and forward genetics approaches (97, 98). Continued innovation in sequencing technologies, host DNA depletion protocols, and bioinformatic tools tailored to *Entamoeba* spp. and other eukaryotic organisms will be essential to furthering protistology research. Moreover, the inclusion of protists in routine microbiome profiling, especially in clinical and epidemiological studies, will be key to redefining baseline health and disease benchmarks across populations.

*Entamoeba* spp. exemplify the need to reframe our understanding of the gut microbiome as a truly cross-kingdom ecosystem. Their roles as immune modulators, microbial sculptors, and potential disease amplifiers are context-dependent and intricately linked to the host and its microbial environment. Recognizing intestinal protists not merely as pathogens, but as ecological participants, opens new avenues for research into gut health, resilience, and therapeutic manipulation. As microbiome science continues to expand its purview, the incorporation of protists into its canon is critical for a more complete understanding of the microbial interactions that shape gut homeostatic and diseased states. The future of gut microbiome research will be richer, more accurate, and more integrative when we embrace these ancient, complex, and often misunderstood inhabitants of the intestinal landscape.
